# Circ_0075829 facilitates the progression of pancreatic carcinoma by sponging miR‐1287‐5p and activating LAMTOR3 signalling

**DOI:** 10.1111/jcmm.16089

**Published:** 2020-11-13

**Authors:** Xudong Zhang, Cailin Xue, Xiaohan Cui, Zhao Zhou, Yue Fu, Xu Yin, Siyuan Wu, Yu Gong, Yi Liu, Chunfu Zhu, Xihu Qin

**Affiliations:** ^1^ Department of Hepato‐Biliary‐Pancreatic Surgery The Affiliated Changzhou No. 2 People's Hospital of Nanjing Medical University Changzhou China; ^2^ Nanjing Medical University Jiangsu China

**Keywords:** Circ_0075829, LAMTOR3, miR‐1287‐5p, pancreatic cancer, tumour malignancy

## Abstract

Pancreatic cancer (PC) is a leading cause of cancer‐related mortality globally. Though increasing evidence has demonstrated that circular RNAs (circRNAs) are linked to the development and progression of cancers, the biological functions of circRNAs in PC remain largely unexplored so far. Based on previous studies, Hsc_circ_0075829 (circ_0075829) was screened out and then further identified in PC clinical specimens and cell lines by real‐time PCR. After the stability tests, a series of in vitro and in vivo functional experiments were performed to investigate the role of circ_0075829 in PC development. Furthermore, fluorescent in situ hybridization (FISH), bioinformatics tools, dual‐luciferase assays and rescue experiments were conducted to clarify the regulatory mechanisms of circ_0075829 in SW1990 and BxPC‐3 cells. Compared with paracancerous tissues, the expression of circ_0075829 was increased in PC tissues, which was positively correlated with the clinical features of PC. Knockdown of circ_0075829 significantly suppressed the proliferative, migratory and invasive rates of SW1990 and BxPC‐3 cells both in vitro and in vivo. Bioinformatics analysis and dual‐luciferase reporter gene assay indicated that circ_0075829 could bind to miR‐1287‐5p. Mechanism research and rescue experiments demonstrated that circ_0075829 could regulate the LAMTOR3/p‐ERK signalling pathway via sponging miR‐1287‐5p in PC cell lines. Our data reveal that the circ_0075829 could facilitate the proliferation and metastasis of PC through circ_0075829/miR‐1287‐5p/LAMTOR3 axis.

## 1 BACKGROUND

Pancreatic cancer (PC) is the fourth cause of cancer‐associated mortality with a lowest overall 5‐year survival rate (<9%) globally.^1^ So far, there have been no clinically sensitive and effective screening services for early‐stage PC. The reality is that more than 80% newly diagnosed patients have already developed locally advanced or metastatic tumours, and surgery is still the first option for treatment.^2^ Despite the significant advances in surgery, chemotherapy and radiotherapy, the therapeutic effects are not ideal for patients due to the chemosensitivity and complex genetic mutations of PC.^3^ Therefore, it is necessary to understand the key molecular mechanisms of this disease and develop new treatment strategies.

Lysosomal adaptor, MAPK and MTOR activator 3 (LAMTOR3, also known as MAPKSP1 or MP1), initially identified as a MEK1/ERK1 scaffolding protein, can regulate ERK1 activation.^4^ Now, LAMTOR3 was recognized as a crucial link in MEK‐ERK interaction and hyperphosphorylation of MEK and ERK. Recent research has demonstrated that LAMTOR3 could activate MAPK and mTOR signalling to improve pancreatic tumorigenesis,^5^, ^6^ making it an important regulatory site in PC.

Circular RNAs (circRNAs), a special type of non‐coding RNAs, are formed by a covalently closed cyclic structure lacking 5′ cap or a 3′ poly A tail. Initially considered as the by‐products of splicing,^7^ circRNAs have been found to play a series of important biological roles such as competing endogenous RNAs (ceRNAs) via sponging microRNAs (miRNAs), protein/peptide transcripts, gene transcription and RNA splicing factors.^8^ In recent years, emerging evidence shows that dysregulated circRNAs could disturb the balance of ceRNA network, and further promote the tumorigenesis of different cancers, including PC.^9^ For example, circ_0061825 facilitates breast cancer progression through targeting miR‐326 and the signal path of its host gene TFF1.^10^ And, circ‐ASH2L is proved to facilitate tumour progression by sponging miR‐34a and affecting the expression of Notch1 in PC.^11^ Meanwhile, some circRNAs may serve as potential biomarkers for cancer diagnosis.^12^ These results indicate that circRNAs are a new category of potential biomarkers or therapeutic targets for PC.

Elevated circ_0075829 (Hsa_circ_0075829) has been identified in PC tissues according to circRNA microarray data.^13^, ^14^ In the present study, we further confirmed the clinical relevance of circ_0075829 expression in PC and discovered that circ_0075829 could promote PC cell proliferation and metastases as a ceRNA to sponge miR‐1287‐5p targeting LAMTOR3. Particularly, the regulation of miR‐1287‐5p/ LAMTOR3 by circ‐0075829 has never been demonstrated before. This article further reveals one regulatory network mechanism of circRNAs in PC, and broadens the horizon for early diagnosis of PC.

## 2 METHODS

### 2.1 Clinical ethics and human tissues

A total of 38 pairs of fresh human pancreatic tissue specimens were obtained from patients who received pancreaticoduodenectomy or distal pancreatectomy at the Affiliated Changzhou No.2 People's Hospital of Nanjing Medical University (China) from February 2017 to May 2019. All patients provided informed consent preoperatively and were followed up regularly after surgery. Patients receiving preoperative neoadjuvant chemotherapy were excluded. This study was approved by the Ethics Committee of the Affiliated Changzhou No.2 People's Hospital of Nanjing Medical University (Registration Number: [2018] KY024‐01). All specimens were pathologically diagnosed.

### 2.2 Cell culture

The human PC cell lines AsPC, PANC1, MiaPaca‐2, SW1990 and BxPC‐3 and normal human pancreatic ductal epithelial cell (HPDE) were obtained from Cellbank of Chinese Academy of Sciences (Shanghai, China). The above cells were cultured in DMEM or RPMI 1640 medium supplemented with 10% foetal bovine serum and 1% penicillin and streptomycin in the incubator (Thermo Fisher, USA) at 37°C with 5% CO_2_.

### 2.3 Real‐time PCR

Total RNA extracted from tissues and cells was prepared by using RNA‐Quick Purification Kit (ES Science) according to the manufacturer's instructions. Extracted RNA was used for reverse transcription to obtain cDNA (Vazyme, USA) (reverse transcription of circRNAs used random primers), followed by RT‐PCR analysis through SYBR Premix Ex Taq™ Kit (Takara). The relative circRNA or mRNA expression level was normalized with β‐actin. U6 was used as internal reference for miRNAs. The microRNA measure kit for miR‐1287‐5p was purchased from Takara (Mir‐X miRNA qRT‐PCR TB Green Kit), and the level of miRNA was tested according to the manufacturer's instructions. The 2^–ΔΔCt^ method was applied to evaluate the expression of target gene. The sequences of primers used in this study are listed in Table S1.

### 2.4 RNase R treatment

Total RNA (2 μg) after being extracted by TRIzol reagent was incubated with 10 U RNase R (Epicentre Technologies, USA) for 20 minutes at 37°C, followed by 70°C for 5 minutes to deactivate the RNase R. After enzyme inactivation, reverse transcription reaction and RT‐PCR can be performed. For controls, the RNA was mock‐treated without the enzyme.

### 2.5 Actinomycin D treatment

SW1990 and BxPC‐3 cells were prepared in a 24‐well plate and exposed to Actinomycin D (2 μg/mL; Sigma) for 0, 4, 8, 12 and 24 hours. After treatment, cells were collected and tested for circ_0075829 and β‐actin expression through RT‐PCR.

### 2.6 Cell transfection

miR‐1287‐5p mimics or inhibitor were purchased from GenePharma Biotechnology (Shanghai, China). Cells were seeded in six‐well plate, and cell density per pore was 50%–60%. After 12 hours, 1 μg non‐specific control (NC), miR‐1287‐5p mimics or inhibitor, 185 μL DMEM and 5 μL Lipofectamine 3000 reagent were mixed to transfect PC cells, and the nutrient medium was changed after 8 hours. Sh‐Circ_0075829 was purchased from Corues Biotechnology (Nanjing, China). The shRNA sequences and the negative control shRNAs were cloned into lentivirus vector GV248 (Corues Biotechnology). All shRNA and miRNA inhibitor sequences are presented in Table S2. Transfection was conducted according to the manufacturer's instructions.

### 2.7 RNA fluorescence in situ hybridization (FISH)

To detect the subcellular location of circ_0075829, FISH was performed with the fluorescent in situ hybridization kit, according to the manufacturer's protocol (RiboBio Biotechnology). The nucleus was counterstained with DAPI. Cy3‐labelled circ_0075829 probes and positive control RNA probes were incubated. The images were obtained with a confocal microscope (Olympus).

### 2.8 Luciferase reporter assay

Circ_0075829 sequence containing the miR‐1287‐5p binding sites was fused into luciferase reporter vector (WT‐Circ_0075829), and then, the site‐directed mutagenesis of the miR‐1287‐5p binding sites in circ_0075829 sequence was inserted into luciferase reporter vector (MUT‐Circ_0075829), following the manufacturer's protocol (RiboBio Biotechnology). 293T was co‐transfected with miR‐1287‐5p mimic or NC together with WT‐Circ_0075829 or MUT‐Circ_0075829 using Lipofectamine™ 3000. Likewise, WT‐LAMTOR3 or MUT‐LAMTOR3 was co‐transfected with miR‐1287‐5p mimic and NC into 293T cells. The luciferase activity was determined 48 hours post‐transfection assaying with Dual‐Glo Luciferase Assay System (Promega) and fluorometer.

### 2.9 Detection of cell proliferation

Cell count kit‐8 (CCK‐8; Beyotime, China) assay, colony formation and 5‐ethynyl‐2′‐deoxyuridine (EdU; RiboBio Biotechnology) assay were utilized to evaluate the proliferation of SW1990 and BxPC‐3 cells. As CCK‐8 assay, stable cells were seeded into 96‐well plates. After incubation for different times (24, 48 or 72 hours), the cells were treated with CCK‐8 reagent for 2 hours. The absorbance of each well (450 nm) was measured in microplate reader. As EdU assay, cells were seeded into 96‐well plates (1‐5 × 10^4^ per well) and then cultured overnight. After 4 hours of incubation in EdU medium, the cells were disposed according to the manufacturer's instructions, and the number of EdU staining cells was counted under the fluorescence microscopy. For colony formation, cells were counted and plated in 6‐well plates (400‐800 per well) and cultured in medium with 10% foetal bovine serum. After 2‐3 weeks, colonies were stained with 0.5% crystal violet. The number of colonies was integrated with ImageJ software to assess cell proliferation.

### 2.10 Wound healing experiment

The cells in active growth phase were seeded in a 6‐well plate. When the cells reached 80% to 90% fusion, a wound was made with a small sterile pipette, and cells were washed lightly with PBS. The scratch width was observed and recorded. After that, the cells were cultured with 2.5% FBS (minimizing the effects of cell proliferation) for 24 hours, the condition of scratch healing was observed and recorded again.

### 2.11 Transwell assay

24‐well transwell (8 μm, Costar, USA) was pre‐coated with Matrigel (BD Biosciences). Cells (1‐5 × 10^4^) were suspended in serum‐free medium and seeded into the upper chamber, and medium supplemented with 20% FBS was applied to the lower chamber. After 12‐24 hours, cells in the top chamber were erased with cotton swabs, and then, cells maintained in the lower surface were fixed with 4% methanol and cells were dyed in 0.5% crystal violet. The random fields were photographed and counted under microscope.

### 2.12 Western blot

Cells were lysed in RIPA lysis buffer with PMSF and protease inhibitor cocktail, the protein concentration was analysed by a bicinchoninic acid kit (Beyotime). Equal amounts of protein were loaded on SDS‐PAGE and transferred to PVDF membrane (Millipore). The membranes were blocked with 5% BSA (Beyotime) and incubated (diluted 1:1000 in buffer) with the following antibodies: LAMTOR3 (CST, #5436), cyclin D (CST, #2978), E‐cadherin (Proteintech, 20874‐1‐AP), N‐cadherin (Proteintech, 22018‐1‐AP), ERK (CST, #6495), p‐ERK (CST, #4370), AKT (CST, #4691), p‐AKT (CST, #4060) and β‐actin (1:2000, CST, #5174) at 4°C overnight. Then membranes were incubated with HRP‐conjugated antibody (1:3000) at room temperature for 1 hour. The target proteins on membranes were detected by ECL kit (Bio‐red). β‐Actin was used for normalization and greyscale values were calculated, respectively. All experiments were performed with three independent trials.

### 2.13 In vivo study

Animal studies were approved by the Ethics Committee of the Nanjing Medical University and performed in accordance with the guiding principles of institutional animal ethics committee. Nude mice (BALB/c; male, aged 4‐5 weeks) were all purchased from Model Animal Research Center of Yangzhou University. To explore cell proliferation, fresh tumour cells in each group (the stable expression cell lines were prepared as described previously, 2 × 10^6^/100 μL per mouse, n = 5 for each group) were injected subcutaneously into the left groin of nude mice. Then, tumour volume was calculated every 5 days (tumour volume = 0.5 × width^2^ × length). 4 weeks later, all nude mice were killed and the tumour specimens were exteriorized and measured. Sections were stained with H&E and IHC. Furthermore, PC cells (1.5 × 10^6^ cells/50 μL) were injected into the pancreas tail of each mouse under anaesthesia for the metastasis model. The vital signs of mice were observed once a week after injection. The tumour implanted in the pancreas in situ and liver metastasis samples were acquired 5 weeks after injection, embedded in paraffin and sectioned. Sections were stained with H&E to assess the extent of metastasis.

### 2.14 Immunohistochemistry (IHC)

For tissue immunohistochemistry, paraffin‐embedded tissues were prepared as stated above, and primary antibody dilutions were as follows: 1:200 Ki67 (CST, #9449) and 1:200 PCNA (CST, #2568). The experiment was completed according to the instructions (Ultrasensitive SP IHC Kit, MXB, China).

### 2.15 Statistical analysis

The statistical analyses were performed with Prism 7 software (GraphPad Software, USA). The results in the bar graphs were presented as mean ± SD. The *P*‐values were calculated by Student's *t* test or ANOVA (analysis of variance), and statistical difference was defined as *P* < .05.

## 3 RESULTS

### 3.1 Circ_0075829 was significantly up‐regulated in PC tissues and cell lines

**Figure 1:**
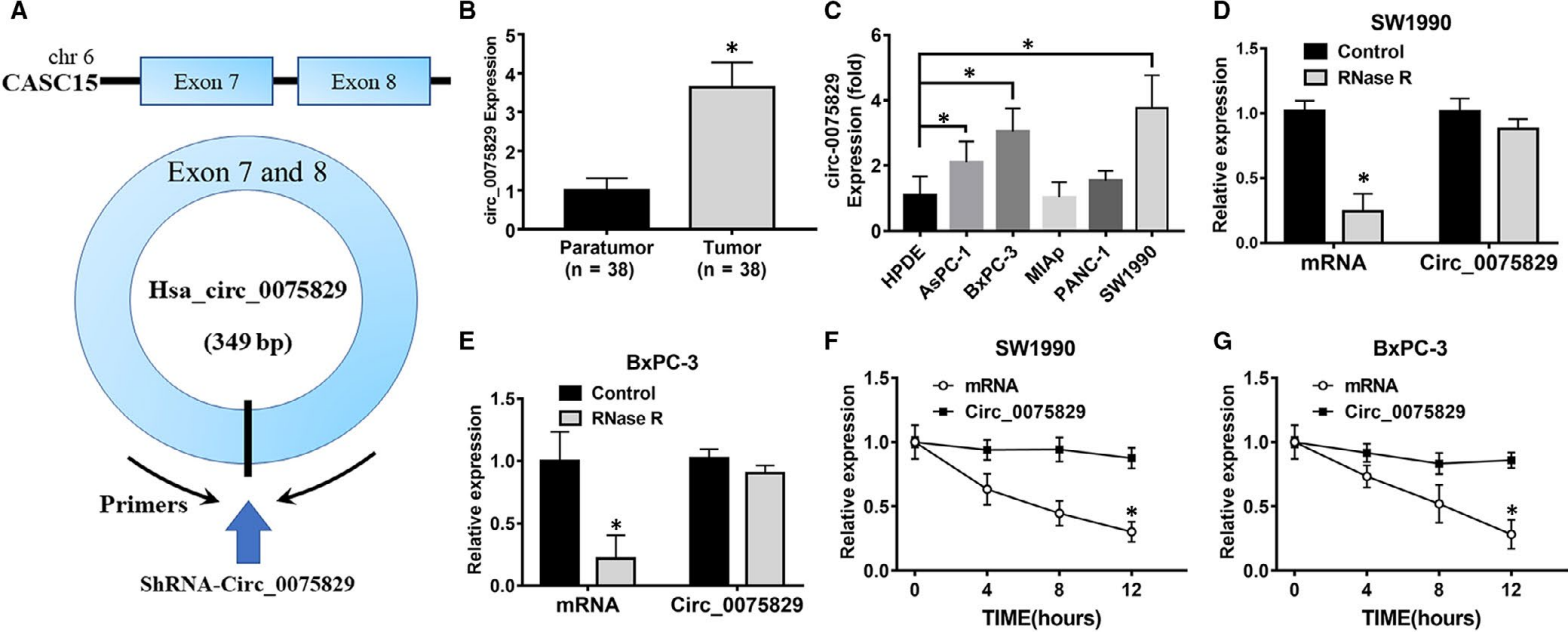
Circ_0075829 was up‐regulated in pancreatic cancer tissues and more stable than linear RNA. A, Details of circ_0075829 and the primer‐designing method of it. B, Circ_0075829 expression in paratumour and tumour tissues from 38 PC patients by RT‐PCR, normalized by β‐actin expression. Data are shown as means ± SD; *P < .05. C, Real‐time PCR analysis of circ_0075829 expression in normal pancreatic cell line (HPDE) and tumour cell lines. D, E, Expression level of circ_0075829 and β‐actin after RNase R treatment of total RNAs in SW1990 and BxPC‐3 cells. F, G, RT‐PCR analysis of circ_0075829 and β‐actin after actinomycin D treatment at the indicated time‐points in SW1990 and BxPC‐3 cells

According to the results of the microarray assay and GEO database in other research, the expression of circ_0075829 was found significantly increased (fold > 2.5, *P* < .001) in PC tissues compared with that in paratumour tissues. Circ_0075829 is derived from exons 7‐8 of the CASC15 gene (chr6:22020567 to 22056919) (Figure 1A). We used 38 pairs of PC and paracancer specimens to examine the expression of circ_0075829. RT‐PCR analysis showed that the expression of circ_0075829 was up‐regulated in PC tumour tissues compared with the adjacent non‐cancerous tissues (Figure 1B). Then, we grouped patients based on their circ_0075829 expression and explored the relevance between the clinicopathological characteristics and increased circ_0075829 expression. The details are summarized in Table 1. Circ_0075829 expression was associated with tumour size (*P* = .017) and regional lymphatic metastasis (*P* = .013). No significant correlation was found between circ_0075829 expression and the patient's age, gender, differentiation and TNM stage. Meanwhile, higher circ_0075829 expression was detected in three PC cell lines (AsPC‐1, BxPC‐3 and SW1990), especially in SW1990 cells, compared with normal pancreatic ductal epithelial HPDE cells (Figure 1C). To elucidate the potential role of circ_0075829 in PC, SW1990 and BxPC‐3 cells were employed in the subsequent studies.

**Table 1:** Clinical characteristics and circ_0075829 expression in patients with PC

	No. of cases	Circ_0075829		*P*
		High	Low	
All patients	38	19	19	
Age				.426
<60	7	5	2	
>60	31	14	17	
Gender				.743
Male	22	10	12	
Female	16	9	7	
Tumour size				.017*
<2 cm	14	3	11	
>2 cm	24	16	8	
TNM stage				.063
I and IIA	10	2	8	
IIB to IV	28	17	11	
Lymphatic metastasis				.013*
Yes	26	17	9	
No	12	2	10	
Tumour location				.269
Head	28	16	12	
Body or tail	10	3	7	
Differentiation grade				.171
I and II	25	15	10	
III and IV	13	4	9	

Note

The data in this table are analysed using Fisher's exact probability method.

* Indicates *P* value < .05.

As circRNAs are characterized by the covalently linked ends, we extracted total RNA from SW1990 and BxPC‐3 cells respectively using RNase R to identify the circular structure of circ_0075829. The β‐actin mRNA expression markedly reduced whereas the circ_0075829 was not, which proved circ_0075829 was resistant to RNase R treatment (Figure 1D,E). We also discovered that unlike linear mRNA, the expression of circ_0075829 was not reduced time‐dependently by actinomycin D treatment in SW1990 and BxPC‐3 cells (Figure 1F,G).

### 3.2 Circ_0075829 promoted PC cell proliferation, migration and invasion in vitro

**Figure 2:**
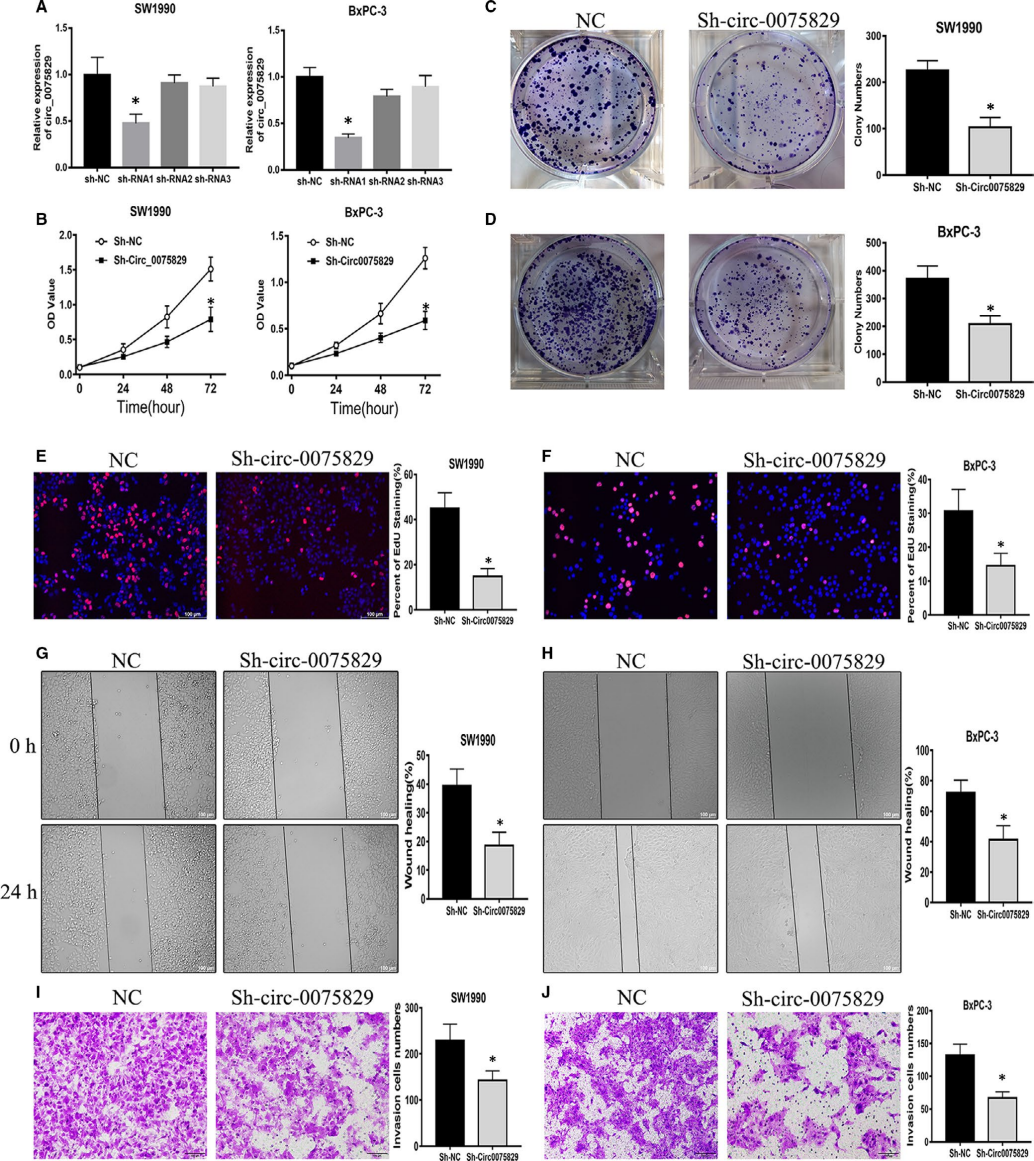
Circ_0075829 promoted cell growth, migration and invasion of PC cells. A, Knockdown efficiency of circ_0075829 by shRNA1, shRNA2 and shRNA3 in SW1990 and BxPC‐3 cells was determined by RT‐PCR, controlled with sh‐NC. B, Cell viability tested by CCK‐8 in SW1990 and BxPC‐3 cells transfected with shRNA1‐circ_0075829 or sh‐NC as indicated above. C, D, Colony formation assay in SW1990 and BxPC‐3 cells treated as indicated. E, F, Cell proliferation analysed by EdU after knockdown of circ_0075829 by shRNA1 in SW1990 and BxPC‐3 cells. G, H, Wound healing assay comparing the migration ability between circ_0075829 knockdown and negative control PC cells. I, J, Transwell assays detecting the changes of cell invasion capacities in SW1990 and BxPC‐3 cells after transfection. Scale bar: 100 μm. All data are shown as mean ± SD (*P < .05). All experiments were repeated at least three times

To analyse the cell functional properties of circ_0075829 in PC, we used circ_0075829‐specific shRNA (Sh‐circ_0075829) targeting the back‐splice junction sequence to down‐regulate the circ_0075829 expression in SW1990 and BxPC‐3 cells. The transfection efficiency was confirmed by RT‐PCR (Figure 2A). Compared with cells transfected with sh‐NC, shRNA1 was selected because it had the most interference efficiency and could not exert significant effects on the expression of host gene‐CASC15 (Figure S1A). CCK‐8 assay (Figure 2B) and colony formation (Figure 2C‐D) revealed that knockdown of circ_0075829 inhibited the proliferation in SW1990 and BxPC‐3 cells. Meanwhile, the results of EdU assay showed that the percentages of EdU dyeing cells were significantly decreased following circ_0075829 knockdown in comparisons with the control groups (Figure 2E,F). As demonstrated by wound healing (Figure 2G,H) and transwell assays (Figure 2I,J), the migration and invasion capabilities of Sh‐circ_0075829‐transfected PC cells were strongly reduced, suggesting that circ_0075829 was also crucial for PC cell migration and invasion.

### 3.3 Circ_0075829 bound directly to miR‐1287‐5p in PC cells

**Figure 3:**
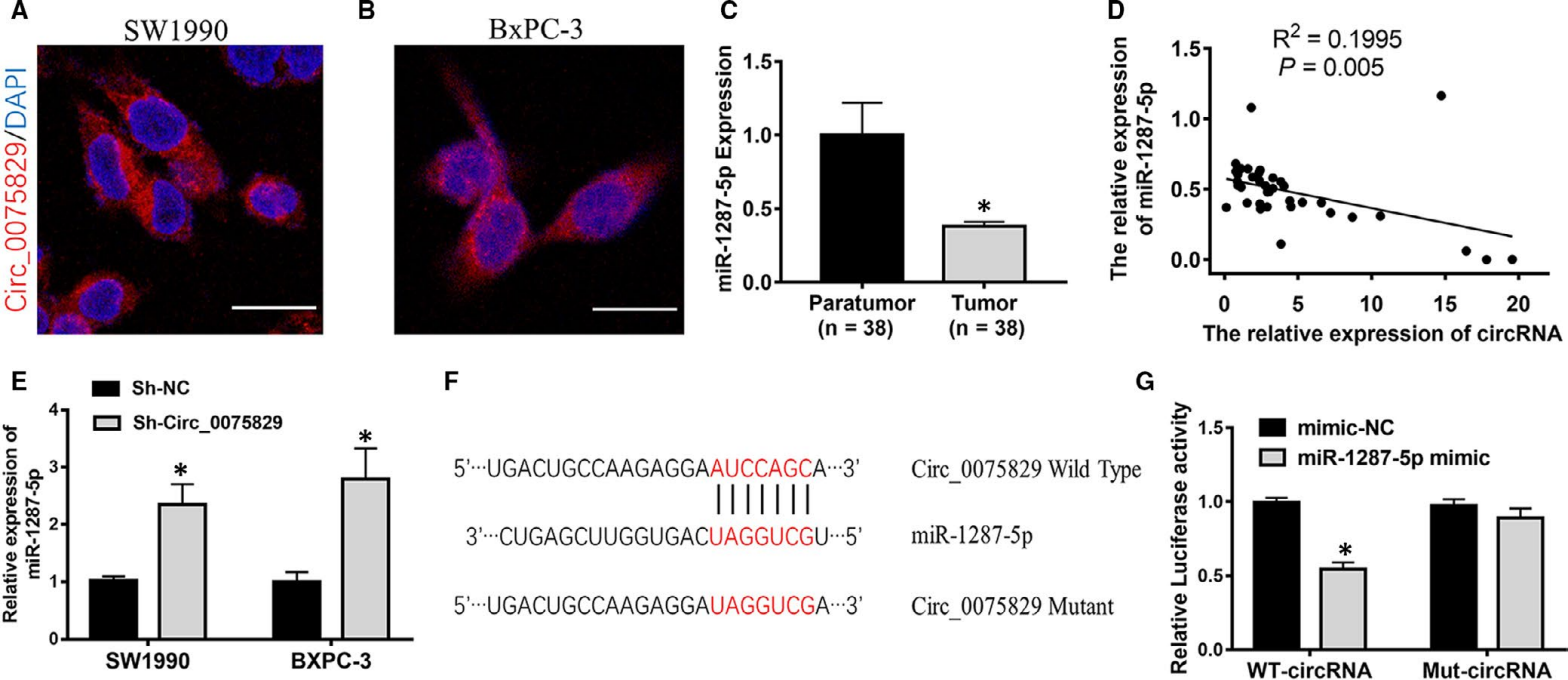
Circ_0075829 functions as a sponge for miR‐1287‐5p. A, B, Localization of circ_0075829 observed in SW1990 and BxPC‐3 cells (magnification, ×400) by FISH. C, Relative miR‐1287‐5p expression in paratumour and tumour tissues from 38 PC patients by RT‐PCR, normalized by U6 expression. D, Pearson's correlation analysis showing the relationship between circ_0075829 and miR‐1287‐5p expression. E, Relative miR‐1287‐5p expression detected after knockdown of circ_0075829 by shRNA1 in SW1990 and BxPC‐3 cells. F, The potential binding sites on circ_0075829 for miR‐1287‐5p mutated for luciferase assay. G, Luciferase reporter assays validated luciferase activity of circ_0075829 reporter in 293T cells co‐transfected with miR‐1287‐5p mimics compared with that transfected with mimic NC (*P < .05)

As for subcellular localization, FISH results showed that most fluorescence signals of circ_0075829 were located in the cytoplasm of SW1990 and BxPC‐3 cells (Figure 3A,B). Previous evidence suggests that circRNAs in the cytoplasm have the potential of sponging miRNAs to regulate gene expression. Two web databases, CircInteractome (https://circinteractome.nia.nih.gov) ^15^ and miRanda (http://www.microrna.org/microrna/home.do), were used to predict the specific miRNAs interacting with circ_0075829. The top 3 intersectional miRNAs, miR‐1287‐5p, miR‐576‐3p and miR‐326, were identified by RT‐PCR (Figure S1B,C). Then, miR‐1287‐5p was selected among the candidate miRNAs, for its low expression in PC and the negative correlation with the circ_0075829 expression as depicted by Spearman's correlation curve (Figure 3C,D). miR‐1287‐5p was highly expressed in both SW1990 and BxPC‐3 cells following the down‐regulation of circ_0075829 (Figure 3E). Thus, we suggested that miR‐1287‐5p was most likely the miRNA sponged by circ_0075829.

Utilizing starBase (http://starbase.sysu.edu.cn/) to predict the miRNA target, we found the putative binding sites of circ_0075829 to miR‐1287‐5p. Then, we constructed the luciferase reporters containing wild‐type 3′UTR sequence of circ_0075829 and the Mut‐circ_0075829 in 293T cells (Figure 3F). The results showed that co‐transfection of miR‐1287‐5p mimics with WT‐circ_0075829‐3′UTR reduced nearly 50% of the luciferase intensity. Conversely, the relative luciferase activity of reporters containing Mut‐circ_0075829 was unaffected by miR‐1287‐5p mimic (Figure 3G). In conclusion, miR‐1287‐5p could be sponged and directly down‐regulated by circ_0075829 in PC cells.

### 3.4 Circ_0075829 regulated LAMTOR3 expression by sponging miR‐1287‐5p

**Figure 4:**
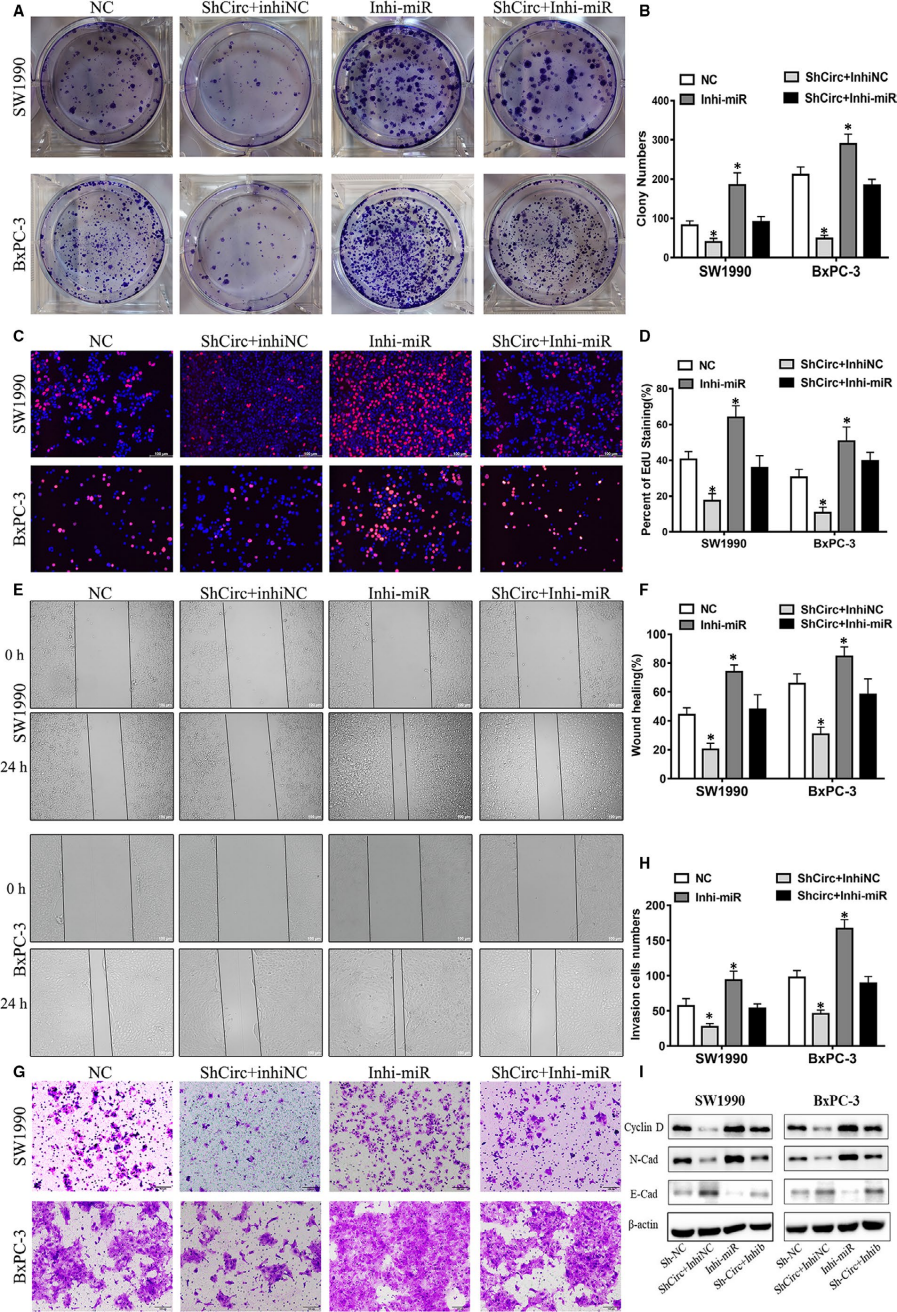
The oncogenic role of circ_0075829 was partly based on its regulation of miR‐1287‐5p. A, B, The cell vitality of NC‐, Sh‐circ_0075829 + inhibitor NC–, miR‐1287‐5p‐inhibitor– and Sh‐circ_0075829 + miR‐1287‐5p‐inhibitor–treated SW1990 and BxPC‐3 cells measured by colony formation assay. C, D, Cell proliferation of SW1990 and BxPC‐3 cells treated as indicated tested by EdU assay. E, F, Cell migration abilities of the above cells measured by wound healing assay. G, H, Cell invasion abilities of the above cells detected by transwell assay. I, Western blot analysis of cyclin D, E‐cadherin and N‐cadherin proteins in the above cell lines, normalized by β‐actin expression. Scale bar: 100μm. Data are from at least three independent experiments and expressed as mean ± SD (*P < .05)

As miR‐1287‐5p has been proved to regulate tumour progression in multiple cancers,^16^ we investigated whether circ_0075829 could promote PC development via miR‐1287‐5p. Through colony formation (Figure 4A,B), EdU (Figure 4C,D), wound healing (Figure 4E,F) and transwell assays (Figure 4G,H), we found the Sh‐circ_0075829–induced suppression of tumour invasion and proliferation could be blocked by miR‐1287‐5p inhibitor in both SW1990 and BxPC‐3 cells, and the same trend was found in proteins related to proliferation and metastasis, such as cyclin D, E‐cadherin and N‐cadherin (Figure 4I).

**Figure 5:**
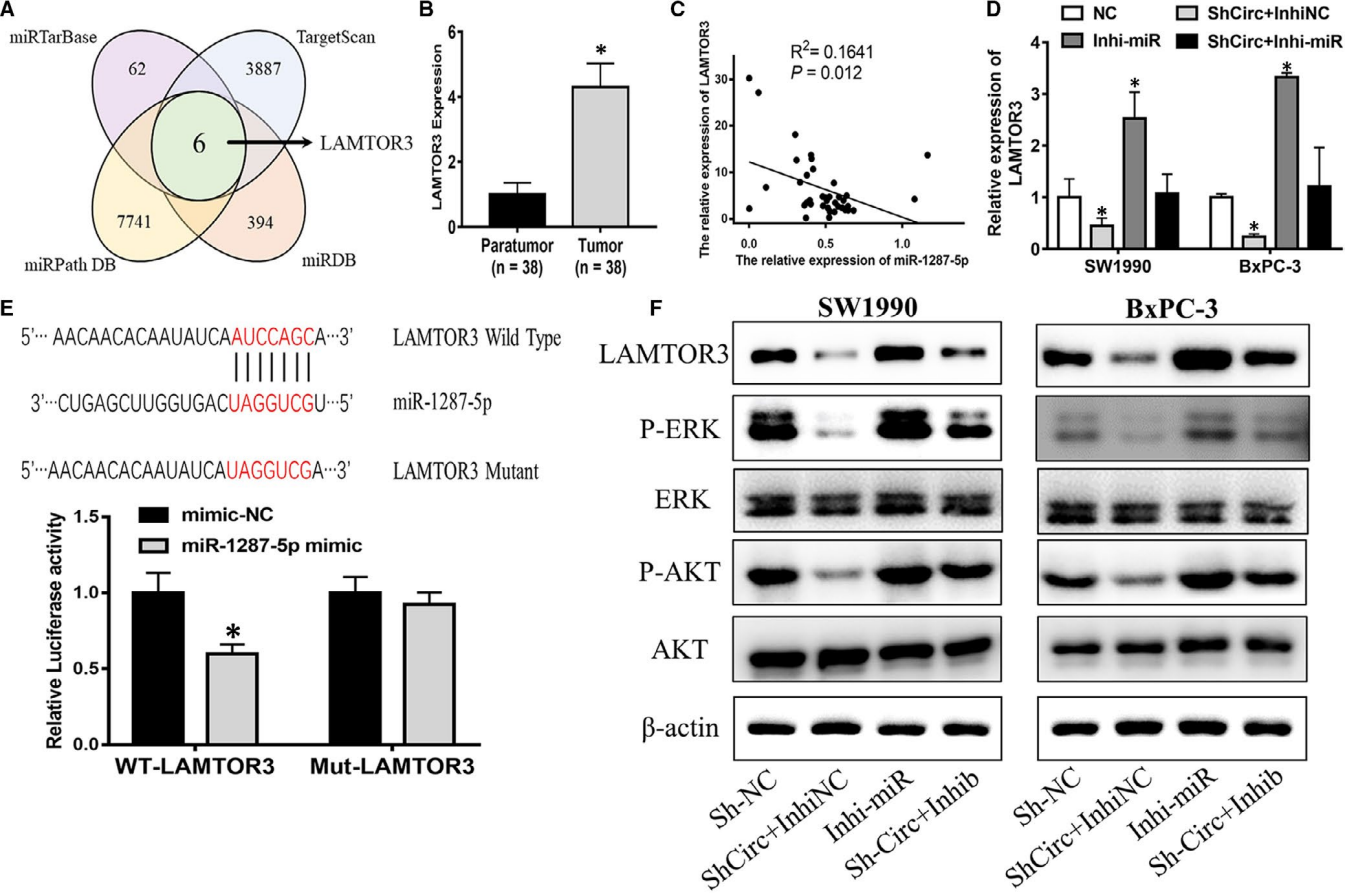
LAMTOR3 expression was stimulated by circ_0075829 through sponging miR‐1287‐5p. A, Six downstream targets (BSCL2, RFX7, LAMTOR3, LONP2, ISY1 and RAB43) of miR‐1287‐5p predicted by bioinformatics tools (TargetScan, miRDB, miRPathDB and miRTarBase) as shown by the Venn diagram. B, LAMTOR3 expression in paratumour and tumour tissues from 38 PC patients by RT‐PCR, normalized by β‐actin expression. C, LAMTOR3 expression negatively associated with miR‐1287‐5p expression in PC samples. D, LAMTOR3 expression level in SW1990 and BxPC‐3 cells after transfection with Sh‐circ_0075829 or co‐transfection with miR‐1287‐5p inhibitor. E, Mutated binding sites on LAMTOR3 for miR‐1287‐5p, and luciferase activity of LAMTOR3‐reporter in 293T cells co‐transfected with miR‐1287‐5p mimics compared with those transfected with NC. F, Western blot analysis for the protein expression of LAMTOR3, p‐ERK, ERK, p‐AKT, AKT in circ_0075829‐knockdown SW1990 and BxPC‐3 cells or cells co‐transfected with miR‐1287‐5p inhibitor or NC. Data are from at least three independent experiments and expressed as mean ± SD (*P < .05)

Utilizing four bioinformatics tools including TargetScan (http://www.targetscan.org/), miRDB (http://www.mirdb.org/index.html), miRPathDB (http://mirtarbase.mbc.nctu.edu.tw) and miRTarBase (http://mirtarbase.mbc.nctu.edu.tw),^17^ we combined four prediction lists and identified 6 potential target genes of miR‐1287‐5p (Figure 5A, Figure S1C). Among them, LAMTOR3 was of particular interest for its high expression in PC and roles regulated by miR‐1287‐5p inhibitor (Figure 5B, Figure S1D). We found the miR‐1287‐5p levels in PC tissue specimens were negatively associated with the expression of LAMTOR3, a gene involved in the MAPK signalling pathway (Figure 5C). Therefore, we speculated that circ_0075829 might regulate LAMTOR3 expression via miR‐1287‐5p. RT‐PCR illustrated that the miR‐1287‐5p inhibitor could increase the LAMTOR3 mRNA expression, whereas the co‐transfection of the miR‐1287‐5p inhibitor and circ_0075829 shRNA could rescue the LAMTOR3 expression both in SW1990 and in BxPC‐3 cells (Figure 5D). A dual‐luciferase reporter assay confirmed that LAMTOR3 was a downstream target of miR‐1287‐5p in 293T cells (Figure S1E, Figure 5E). Western blot assay results showed the same tendency of LAMTOR3 and downstream protein factors such as p‐ERK and p‐AKT (Figure 5F). These findings illustrated that circ_0075829 could participate in PC development by activating LAMTOR3 via miR‐1287‐5p.

### 3.5 Circ_0075829 promoted tumorigenicity and metastasis in vivo

**Figure 6:**
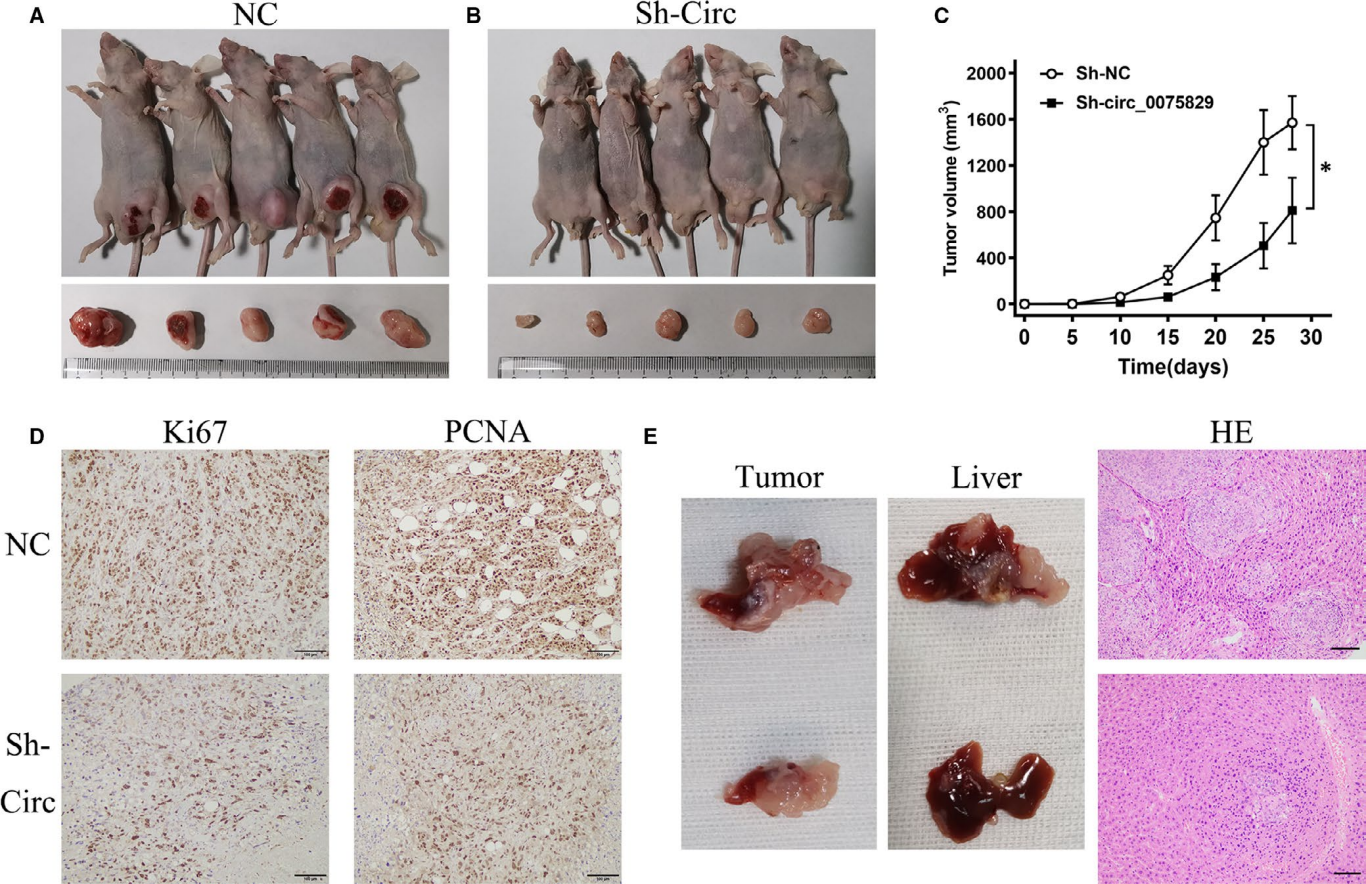
Circ_0075829 promoted tumour growth and metastasis in PC cells in vivo. A, B, Tumours of mice xenograft executed at day 28 after injection with Lv‐Sh‐NC (SW1990) and Lv‐Sh‐circ_0075829 (SW1990). C, Subcutaneous tumour growth curve of xenograft mice injected with indicated cells. D, Immunohistochemistry staining of Ki67 and PCNA in mouse subcutaneous tumours after silencing circ_0075829. E, Pancreatic tail xenograft models established to show the role of circ_0075829 in metastasis in vivo. Primary tumour xenografts, liver metastases by autopsy and corresponding H&amp;E staining of circ_0075829 knockdown and NC groups are shown representatively (*P < .05, scale bar, 100 μm)

The above results indicated the important role of circ_0075829 in tumour progression in vitro. To further inquire the role of circ_0075829 in vivo in PC progression, we established the subcutaneous xenograft tumour model and the pancreas tail injection model. SW1990 cells transfected with Lv‐Sh‐circ_0075829 were injected into the inguinal subcutis of 4‐week‐old male nude mice to generate xenografts. During the 1‐month experiment, the size of the subcutaneous tumour was measured regularly. Figure 6 A‐C shows that subcutaneous tumours grew slower and smaller in the Lv‐Sh‐circ_0075829 injected group. From IHC, the proliferation‐related protein, Ki67 and PCNA, presented lower positivity after silencing circ_0075829 (Figure 6D, Figure S1F). Furthermore, for pancreatic tail injection with SW1990, gross specimens and HE staining showed that circ_0075829 knockdown both decreased the size of in situ implantation and ameliorated liver metastasis compared with the control group (Figure 6E, Figure S1G). Hence, all the above observations indicated that circ_0075829 promoted PC growth and metastasis in vivo.

## 4 DISCUSSION

In the 1970s, circRNAs were first observed in the genome of viruses and in the cytoplasm of eukaryotic cells.^7^ Given that circRNAs were less susceptible to exonuclease digestion than linear ones due to their covalently closed circular structure, scientists have been working on their biological functions for decades. Recently, increasing evidence has demonstrated that circRNAs could act as miRNA inhibitors ‘sponges’ and protein ‘decoys’, or encode small peptides directly.^18^ As miRNA sponges, circRNAs could sequester miRNA away from its target mRNAs and then relieve its mediated gene suppression. miRNAs, the small non‐coding RNAs of 18‑25 nucleotides, play momentous roles in post‐transcriptional modification of gene expression by specifically targeting mRNAs. For instance, circANKS1B could promote colorectal cancer cell invasion by functioning as a sponge of miR‐149 to regulate the FOXM1 expression.^19^ Meanwhile, exosome‐delivered circRNA_0005963, as a sponge for the PKM2‐targeted miR‐122, was positively correlated with chemoresistance in colorectal cancer.^20^ Identified through circRNA pull‐down and mass spectrometry, circRNAs could also bind to and then sequester proteins, serving as protein decoys or scaffolds to regulate gene expression.^21^ For example, circFOXK2 was found to complete with Y‐box binding protein 1 and heterogeneous nuclear ribonucleoprotein K to promote the expression of oncogenic proteins such as pyridoxal kinase in PC.^22^ Some circRNAs have the open reading frame (ORF) and ribosome binding site for translation of regulatory peptides, suggesting their potential of translation. Ribosomal profiling, as a bioinformatics tool to monitor translation in vivo by sequencing ribosome‐covered RNAs, has provided convincing evidence of circRNAs translation.^23^ Therefore, as a new class of RNAs with tissue/development‐specific expression patterns, circRNAs, commonly dysregulated in a variety of malignancies, are involved in the development of various carcinomas.

In the current study, by integrating two previous studies, we screened out circ_0075829, which was highly expressed in PC. Clinically, the overexpression of circ_0075829 was correlated with the lymphatic metastasis and tumour size of PC, which arised our interest in its role in PC development. After shRNA interference, circ_0075829 was found to promote PC cell proliferation, migration and invasion. Considering that most circRNAs in the cytoplasm were functioning as miRNA sponges, we used bioinformatics analyses to predict the potential RNAs that might bind to the 3′UTR of circ_0075829, and proved circ_0075829 containing the binding sites for miR‐1287‐5p using luciferase reporter assays. Then, RT‐PCR results also showed the negative correlation between the expression of circ_0075829 and miR‐1287‐5p in PC tissues. Therefore, we confirmed that circ_0075829 reduced miR‐1287‐5p expression by serving as a ceRNA. miR‐1287‐5p could suppress the tumour by targeting various genes in different cancers including breast, lung and cervical cancer,^24^, ^25^, ^26^ and was also found to be a target of circRNAs. For example, circSLC26A4 could accelerate cell proliferation and invasion via absorbing miR‐1287‐5p/HOXA7 in cervical cancer.^27^ However, the role and mechanism of miR‐1287‐5p in PC have not been reported before. Our research first discovered that interfering with the miR‐1287‐5p expression could significantly increase the proliferation and metastatic ability of pancreatic tumour cells. Meanwhile, a series of rescue experiments showed that the inhibition of miR‐1287‐5p could reverse the inhibitory effects of circ_0075829 knockdown on PC cell proliferation and motility, which was also present in the proteins related to proliferation and metastasis, such as cyclin D, E‐cadherin and N‐cadherin. To elucidate the downstream factors of the circ_0075829/ miR‐1287‐5p pathway in PC progression, we predicted that miR‐1287‐5p might be related to the expression of LAMTOR3 using the bioinformatics analysis and PCR. LAMTOR3 dysregulation was not only found in PC,^28^ glioma,^29^ breast cancer ^30^ and gastric cancer,^31^ but also linked to proliferation, metastasis and cell differentiation.^32^ We found that LAMTOR3, as a scaffold protein between MEK1 and ERK1, was the target of circ_0075829/miR‐1287‐5p in PC progression, because the inhibition of miR‐1287‐5p could partially reverse the decline in LAMTOR3/p‐ERK caused by the knockdown of circ_0075829. Combining with subsequent in vivo experiments of circ_0075829, we manifested that circ_0075829 could affect the proliferation and metastasis of PC cells by targeting miR‐1287‐5p to regulate LAMTOR3/p‐ERK expression.

For the first time, the functions and interactions of circ_0075829 and miR‐1287‐5p in PC were uncovered. However, the studies of circRNAs in PC are still at initial stage. PC progression essentially depends on the crosstalk with the tumour microenvironment. With the help of genetic mouse models of PC, further circRNA research may focus on the PC tumour microenvironment, such as macrophages and NK cells.^33^ Relying only on bioinformatics analysis to further excavate downstream pathways has certain limitations. It has also been reported that single circRNA can sponge up multiple types of miRNAs.^34^ Using single‐cell sequencing or RNA pull‐down assay, more multivariate and in‐depth circRNA/miRNA regulatory mechanisms in tumour microenvironment would be discovered. Based on the high stability of circRNA, non‐invasive detection in body fluids may be the future direction of their clinical transformation.^35^ For the correlation between circRNA and PC survival, more long‐term follow‐up data and more accurate clinical feature grouping are needed. A better understanding of circRNAs is momentous for a better comprehension of PC pathological process. As LAMTOR3‐guided ERK and AKT are both important downstream signal pathways of K‐Ras, the oncogenic mutation is present in 90% of pancreatic neoplasms, as well as a research hot spot of drug therapeutic target.^36^, ^37^ Research into the convergent roles of circRNAs in K‐Ras signalling pathway might propose a new insight into PC treatment. However, there is still a long way to go before the clinical application of circRNAs.

## 5 CONCLUSION

Taken together, this study unveiled the critical function of circ_0075829 in PC progression. CircRNA_0075829/miRNA‐1287‐5p/LAMTOR3 regulates PC tumorigenesis. Therefore, our data suggest the considering circRNA‐based diagnostic strategies for PC.

## CONFLICT OF INTEREST

The authors confirm that there are no conflicts of interest.

## AUTHOR CONTRIBUTIONS

**Xudong Zhang:** Conceptualization (equal); formal analysis (equal); methodology (equal); project administration (equal); writing‐original draft (lead). **Cailin Xue:** Data curation (equal); formal analysis (equal); methodology (equal). **Xiaohan Cui:** Data curation (equal); formal analysis (equal); methodology (equal). **Zhao Zhou:** Methodology (equal); validation (equal). **Yue Fu:** Methodology (equal); validation (equal). **Xu Yin:** Data curation (equal); visualization (equal). **Siyuan Wu:** Data curation (equal); visualization (equal). **Yu Gong:** Data curation (equal); visualization (equal). **Yi Liu:** Data curation (equal); formal analysis (equal). **Chunfu Zhu:** Conceptualization (equal); project administration (equal); writing‐review and editing (equal). **Xihu Qin:** Conceptualization (equal); funding acquisition (lead); project administration (lead); writing‐review and editing (equal).

## Supporting information

Fig S1

Table S1‐S2

## Data Availability

The data that support the findings of this study are available on request from the corresponding author. The data are not publicly available due to privacy or ethical restrictions.
