# Supplementary figures and images for: Circ_0075829 facilitates the progression of pancreatic carcinoma by sponging miR‐1287‐5p and activating LAMTOR3 signalling
Source: J Cell Mol Med. 2020 Nov 13;24(24):14596–607. doi: 10.1111/jcmm.16089 (PMC7753824; doi:10.1111/jcmm.16089)

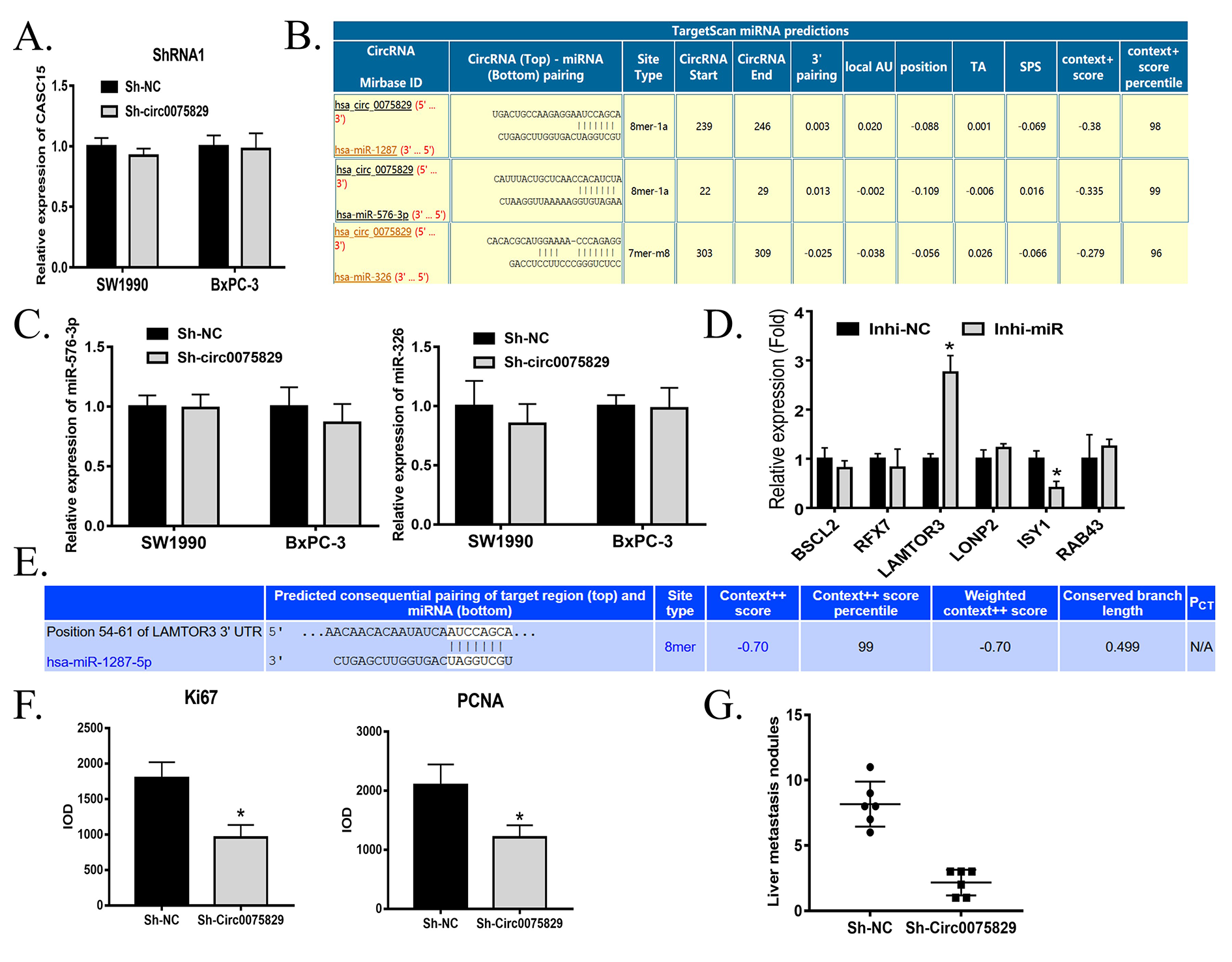

Supplement: Supplementary file 1 — Fig S1 [file JCMM-24-14596-s001.tif]
