# Supplementary material for: Circ_0075829 facilitates the progression of pancreatic carcinoma by sponging miR‐1287‐5p and activating LAMTOR3 signalling
Source: J Cell Mol Med. 2020 Nov 13;24(24):14596–607. doi: 10.1111/jcmm.16089 (PMC7753824; doi:10.1111/jcmm.16089)
Supplement: Supplementary file 2 — Table S1‐S2 [file JCMM-24-14596-s002.docx]

**Supplementary Table S1. Primers for Quantitative RT- PCR**

| **Gene name** | **All Patients** | **Sequence（5’---3’）** |
| --- | --- | --- |
|  |  |  |
| hsa_circ_0075829 | Forward Primer | AAGAGAGCCAGCCAGGATCTG |
|  | Reverse Primer | AGAAGGATGTTCAGTAGTAACCCAG |
| CASC15 | Forward Primer | CAAGAGGAATCCAGCAAAGC |
|  | Reverse Primer | CATGGAGAGAGGACCTGAGC |
| β-actin | Forward Primer | GTCTTCCCCTCCATCGTG |
|  | Reverse Primer | AGGGTGAGGATGCCTCTCTT |
| U6 | Forward Primer | CTC GCTTCGGCAGCACA |
|  | Reverse Primer | AACGCTTCACGAATT TGCGT |
| LAMTOR3 | Forward Primer | ATGGCGGATGACCTAAAGCG |
|  | Reverse Primer | ATGGAGCCCTTCAACACTTGG |
| miR-1287-5p | Forward Primer | AGCTGGATCAGTGGTTCGAG |

**Supplementary Table S2. Summary of Transfection Oligos**

| **Name** | **Oligo Sequence** |
| --- | --- |
| Circ_0075829 shRNA 1 | GATCCGAGAGCCAGCCAGGATCTGCATCA  AGAGTGCAGATCCTGGCTGGCTCTCTTTTTT |
| Circ_0075829 shRNA 2 | GATCCGAGCCAGCCAGGATCTGCATTTCAA  GAGAATGCAGATCCTGGCTGGCTCTTTTTT |
| Circ_0075829 shRNA 3 | GATCCGCCAGCCAGGATCTGCATTTATCAA  GAGTAAATGCAGATCCTGGCTGGCTTTTTT |
| Sh-NC | GGATCCGTTCTCCGAACGTGTCACGTTTCA  AGAGAACGTGACACGTTCGGAGAATTTTTT |
| MiR-1287-5p inhibitor | GACUCGAACCACUGAUCCAGCA |
| Inhibitor NC | CAGUACUUUUGUGUAGUACAA |
| MiR-1287-5p mimics | UGCUGGAUCAGUGGUUCGAGUC |
